# Industry Perspective – What does Industry Need to Accelerate Drug Product and Process Development?

**DOI:** 10.1007/s11095-023-03604-y

**Published:** 2023-10-11

**Authors:** Bikash Chatterjee, Richard Steiner, Goldi Kaul

**Affiliations:** 1 Pharmatech Associates - a USP Company, Hayward, CA USA; 2 External Alternative CMC Development, Boehringer Ingelheim Pharmaceuticals, Ridgefield, CT USA

**Keywords:** accelerating, ASAP testing, bayesian, continuous manufacturing, data, drug substance, *in-silico* modeling, modeling

At the National Institute for Pharmaceutical Technology & Education (NIPTE) pathfinder workshop held at The Bush School of Government & Public Service in Washington D.C., a high-ranking group of leaders from academia, industry, and the FDA met to discuss ways to accelerate drug product development and approval in light of critical patient needs and the national emergency of drug shortage and supply chain risks.

Two clear objectives were achieved:

- To identify Scientific, Technological, and Regulatory barriers that prevent significant reductions in the drug discovery, development, and commercialization time frame.
- To pinpoint mechanisms capable of significantly reducing the time required for drug discovery, development, and commercialization, and integrate these into a coherent strategy and implementation plan to improve efficiency and reduce risks to patients.

The rapid development, approval, and success of the COVID-19 vaccines raises the prospect of applying lessons learned there going forward: streamline and expedite the drug development process without impacting health authority, drug sponsor and patient confidence in the safety and efficacy of new drug therapies. The industry has embarked on a renaissance of innovation with 37 novel drug therapies approved in 2022, 20 of which were first-in-class drug therapies with completely unique mechanisms of action (https://www.fda.gov/drugs/new-drugs-fda-cders-new-molecular-entities-and-new-therapeutic-biological-products/new-drug-therapy-approvals-2022). The question for this working group: how to get these therapies faster to patients who need them? A recent study (https://lifesciences.n-side.com/blog/what-is-the-average-time-to-bring-a-drug-to-market-in-2022) determined that it required between 10–15 years to go from molecule discovery to FDA approval with an approximate development cost of $2.6 billion per drug therapy. On average, a drug sponsor will spend 2.3 years in Phase I; 3.6 years in Phase II; 3.3 years in Phase III; and 1.3 years between Phase III and regulatory approval. For an industry with a 12 percent success rate for drugs entering clinical trials (https://www.cbo.gov/publication/57126#:~:text=Developing%20new%20drugs%20is%20a,for%20introduction%20by%20the%20FDA) and subsequently being brought to market, the ability to quickly and cost-effectively move through the drug development process and eliminate potentially unsuccessful candidates early in the drug development lifecycle can impact time to market for successful therapies and also the cost of drug therapies in the marketplace.

## Overcoming Barriers to Acceleration

**Fig. 1:**
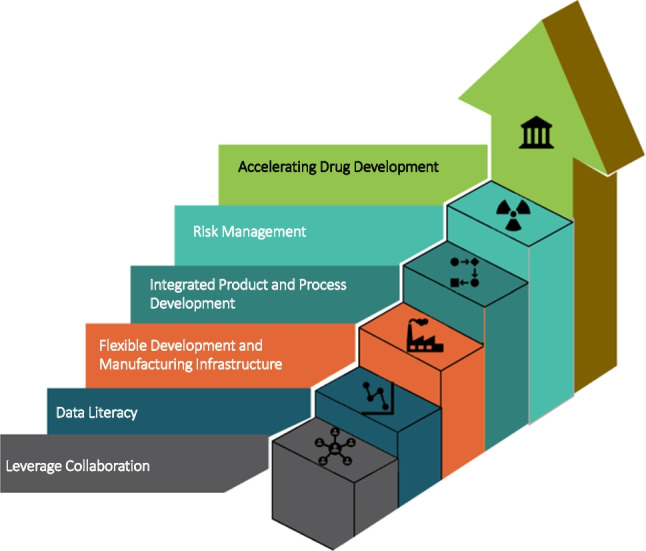
Industry barriers to accelerating drug development.

Barriers to accelerating the drug development timeline subsume two basic elements – barriers that represent greater efficiency because of advances in technology; and barriers that require a paradigm shift in regulatory and scientific thinking. The workshop identified the following five areas (Fig. 1) as fundamental barriers to realizing acceleration from an industry perspective:

## Risk Management

While risk management has been a part of most industries for many years it didn’t really gain traction in the pharmaceutical industry until the issuance of ICH Q9 in 2006 and the issuance of the 2011 Process Validation guidance in the U.S. Today, risk assessments are part of many key steps of the drug development lifecycle and are a core component of any regulatory argument and evaluation framework. Dealing with uncertainty has always been a key consideration in drug development. Whether justifying a sampling plan or powering a clinical study, quantifying uncertainty is a foundational component of drug development decision making.

### Statistics: Frequentist *vs.* Bayesian Thinking

There are two schools of thought when it comes to decision making: the Frequentist approach and the Bayesian approach. The Frequentist approach to statistics makes predictions on the underlying truths of the experiment, using only data from the current experiment**.** This thinking underpins most statistical decision making in drug development. The Bayesian approach to statistics by contrast is based on encoding past knowledge of similar experiments into a statistical device, known as a *prior*. This prior is combined with current experiment data as part of the decision-making process. A fundamental aspect of Bayesian inference is updating your understanding in light of new evidence. Bayesian inference recognizes the value of both experience and expertise in arriving at a decision, while the Frequentist approach does not. Industry has long sought to leverage knowledge from previous development activities as a basis for managing product development uncertainty.

There are multiple examples of FDA recognizing the value of Bayesian inference in managing risk. The 21st Century Cures Act (https://www.congress.gov/bill/114th-congress/house-bill/34.) of 2016 was designed to accelerate medical product development and bring new innovations and advances faster and more efficiently to the patients who need them. The act specifically called for the incorporation of Real-World Evidence (RWE) as a driver for accelerating the approval process. In 2018, FDA created a framework for evaluating the potential use of RWE to support the approval of a new indication for a drug already approved under Sect. 505(c) of the FD&C Act, or to support or satisfy drug post-approval study requirements. The same underlying principle exists in the FDA’s breakthrough, orphan, rare disease and fast-track designation programs. However, it is the application of the FDA’s Emergency Use Authorization (EUA) Program during the development of the COVID-19 vaccines that proved the need and benefits of adopting a Bayesian approach to speed drugs to market without compromising safety and efficacy.

The EUA program was established in 2004 as part of the BioShield Act as a result of the anthrax scares of the early 2000s. It requires a formal declaration of a national emergency to trigger the EUA process. Comparing the EUA process for the Moderna and Pfizer vaccines to the approval process for Merck’s Gardasil, the HPV vaccine, the differences could not be more stark. Moderna began initial development 1/23/2020 (three days after first U.S. COVID-19 case was identified). The first patient was dosed for Moderna Phase 1 (3/16/20) and for the Pfizer/BioNTech collaboration (3/17/20). The EUA was announced 3/27/20. Both companies applied for EUA at the end of November 2020. On 1/21 the first doses were released commercially, a mere 12 months since the program began. By contrast, work on Gardasil (Merck) was initiated in 1992 with the first Phase 1 dose in 1997. Final FDA approval was obtained in 2006, nine years after the first human dose. The COVID vaccines were approved based upon the totality of scientific evidence available (including clinical trials, if available) and based on the known benefits of taking the product outweighing the known risks of the product. FDA intelligently managed the risk without compromising efficacy and safety.

The agency was very effective in developing and executing the EUA process during the pandemic. This raises the question if similar thinking could be applied in a broader context to drug development that would accelerate the approval of new therapies.

For example, could there be an intermediate designation process that doesn’t rise to the level of EUA but moves more quickly than the typical BLA/NDA? A key attribute of the EUA framework was the close collaboration between drug sponsors and FDA during the development of the vaccines. This provided needed clarity in a much more effective framework than the current Type C meeting or WRO framework, resulting in greater development efficiency. For example, could a regulatory framework applied to breakthrough, orphan, rare diseases with fast-track designation be more broadly applied to the traditional development framework? The agency has accepted the validity of biomarkers and other clinical intermediates endpoints as a surrogate for fully defined mechanisms of action, a core catalyst for the development and approval of new complex large molecule drug modalities in the immuno-oncology and cell and gene therapy area. How could this same concept be applied in other areas (e.g., product performance, other development activities)? In the absence of a full model, this might create agility to negotiate a more rapid path to demonstrating what’s needed for approval. These frameworks could also incentivize drug sponsors who are reluctant to engage in dialogue with FDA during the development process, avoiding additional cost for drug sponsors because of unexpected delays while bringing drug therapies to patients faster. Bringing drugs to the market more efficiently through the targeted use of science and experience benefits drug sponsors, patients, and the FDA. As the industry grapples with the repercussions of the Inflation Reduction Act and looks to reevaluate portfolios of lower revenue small molecule programs, a targeted accelerated pathway, which provides a lower cost of development may be the offset all parties need to see a solution that benefits all involved,

### Complementary Characterization: Stability Testing

Stability testing is one step in the drug development cycle that requires significant time to prepare for and execute. The IQ Consortium has gathered survey data from drug sponsors regarding drug filing successes and failures over a five-year period and found that 50 percent of the programs had less than 12 months of stability data at filing. Could a Bayesian approach reduce risk for both drug sponsors and regulators alike? For example, could a shorter stability study be bolstered by prior knowledge of a similar molecule or dosage form data that has been modeled to predict behavior on stability? If combined with a commitment by the drug sponsor to complete real time stability testing this could move this critical step to a parallel activity instead of making it a prerequisite to filing, and shave years off the development time.

Historically the FDA has allowed drug sponsors to use accelerated testing during development. Products are tested under accelerated conditions to increase the rate of chemical and/or physical degradation. Based on the Arrhenius equation the chemical degradation increases with the temperature and therefore it should be possible to project the degradation rate at low temperature from the data generated under accelerated or stressed conditions. However, the predictive power of those experiments is not always sufficient, hence the FDA still requires real time stability data for its filing. Part of the challenge with the predictive power of Arrhenius equations is they only consider temperature as a variable in their design when many drug modalities have other factors that can influence degradation, such as humidity or percentage oxygen.

One approach which has demonstrated superior predictive power over the classical accelerated testing is the **Accelerated Stability Assessment Program** (ASAP). With ASAP, instead of keeping time points fixed and determining the amount of degradant/potency change as you would with a conventional Arrhenius plot, isoconversion keeps the amount of degradant/potency change constant at the specification limit and varies the time. ASAP studies are quick and can be completed in about a week. Complementing real time stability data with an ASAP data set could provide significant assurance of stability, allowing programs to move forward until confirmatory real time data is available.

Adopting the FDA’s approach to **Physiologically Based Pharmacokinetic Modeling** (PBPK), it could be possible for industry and the FDA to partner with academia to develop predictive stability models for drugs that have shown no history of issues around stability, to determine how similar molecules would demonstrate similar risks. The goal of such a framework would be to define alternative pathways for drug sponsors that could address FDA’s concerns in lieu of real time data, in effect making the expectation of more data the exception rather than the rule.

## Integrated Process and Product Development

Building upon the notion of risk reduction through complementary or predicate data sources as a surrogate for product specific data, significant acceleration could be achieved during development if predicate knowledge of similar molecules is used as basis for moving forward during drug development. FDA has already planted the seeds of this concept in its biowaiver process. For BCS 1 (highly soluble-highly permeable) API molecules as part of an immediate release (IR) dosage delivery form, the agency has clearly articulated what the criteria would be to ask and qualify for a biowaiver. The result can shave 12–18 months from the development timeline for a generic drug today and greatly simplify the review process as well.

Similarly, the FDA modernization act (https://www.congress.gov/bill/117th-congress/senate-bill/5002) which passed in December 2022, decreed that FDA no longer would require animal testing for a drug to be approved. The motivation for change is the imperfectness of the animal testing model— where more than 90 percent of all drugs that pass initial animal tests end up being unsafe or ineffective in humans [1]. Industry is just as cautious as the FDA in making changes and the shift to animal testing surrogates. For FDA to promote the adoption of new tools, such as AI-based models that predict toxicity in particular organs, or surrogate testing frameworks such as organoid or spheroids as used in oncology applications to model tumor behavior, they would have to participate in defining the criteria for model validation and what is good enough, while acknowledging and recognizing the poor performance of the predicate animal testing model that has been the standard for all IND approvals.

Couldn’t a similar model be extended beyond just the same molecule for new chemical entities (NCEs)? For example, if industry were to develop a model(s) based upon well-defined characteristics of an API such as chemical structure, molecular weight, nature of the drug substance (acid, base, amphoteric or neutral), and dissociation constants (pKa), could FDA define a set a of criteria to allow industry to take advantage of cumulative understanding of similar molecules, allowing them to move ahead on risk with less stability and toxicology data? There would be commitment to do real time testing in parallel as the programs move forward through registration lots, to cut years from the development program. The tools to develop and control these models are evolving rapidly as other industries look to harness the predictive power of predicate data. It would be an easy exercise for industry and FDA to collaborate and define the key characteristics of the model required, to give confidence in its capabilities.

The advancement of in silico modeling and “organ on a chip” represents significant opportunity for biologics and small molecules alike potentially simplifying and accelerating safety studies while laying the groundwork for moving to PBPK modeling for First in Human (FIH) studies in lieu of clinical studies. This would shave off considerable cost and time from the Pre-IND-IND timeline.

Industry has some control over realizing these improvements. Adopting an approach such as **formulation by design** in which formulations are developed with the downstream process in mind is worth considering. Industry has begun to move toward this thinking by delaying the selection of the final dosage form until Phase 2 to avoid the challenges sometimes encountered with dosage formats that are constrained by the API’s or Drug Substance’s toxicological ceiling. Formulation by design expands this approach to consider commercial processability at the product design stage and as part of the Product Technical Product Profile (TPP). For example, applying principles of quality by design (QbD) to the selection of excipients along with downstream processability considerations could dramatically reduce process variability as the process is scaled up. All of these iterative improvements would have a very positive impact on shortening time to market while simplifying both the process development and regulatory review process.

## Flexible Development and Manufacturing Infrastructure

The notion of doing things better, faster, and cheaper has always been attractive to industry. The industry has seen the advent of a number of innovations that fall under the umbrella of **Advanced Manufacturing Technology** (AMT) that present significant opportunities in both risk reduction during development and in shortening time to market. AMT represent a step-function improvement to the control and understanding of how drugs can be manufactured. They demonstrate a commensurate improvement in overall drug quality yet adoption by industry has been slow. Within industry and FDA, AMTs such as pharmaceutical continuous manufacturing (PCM) are poised to gain broader adoption. PCM represents the next evolution in manufacturing for pharmaceutical and biologic drug manufacturers, eliminating intermediate storage and release of products and their associated quality issues, simplifying process development as there is no scale up required between development and commercial, while presenting the opportunity for lower costs and higher yields through greater control and monitoring of the process. Technological barriers associated with equipment design and compatibility, PAT and the complexity of data analysis are rapidly being addressed by suppliers and industry alike. As these elements move toward common standards and solutions, the adoption of AMT such as PCM will move beyond innovator companies to generic and OTC drug manufacturers, thus providing the industry with not only greater process control and product quality but also arming them with a powerful new tool for driving business performance- a win–win situation for regulators, industry and patients alike.

Large molecules are also actively looking at targeted implementation of continuous manufacturing to realize the benefits of great product quality and yield through in-line control along with improved operating efficiency in upstream or downstream processes.

Organizations that have implemented PCM have provided clear feedback on what it takes to move to a new platform and effect a paradigm shift in thinking through a drug sponsor. Once a drug sponsor believes they have a plan to adopt and gain proficiency, it takes a strategic objective at the executive or board level, such as getting drugs to patients more rapidly, coupled with highlighting the “game changing” advantages of adoption. FDA can bolster this with a clear roadmap for drug development complemented by training compliance inspectors in the advantages for surveillance and pre-approval inspections. The reality is there will be less of the traditional quality issues with new AMTs –a win for health authorities and industry. Training for compliance inspectors and training for industry is essential to spur broader adoption by industry.

## Data Literacy

Data literacy must become an industry core competency if industry and FDA are to realize a broader adoption of AMTs, move toward Bayesian principles to drug development, and realize improved drug quality and speed to market. As other industries embrace the principle of Industry 4.0, the foundational components of acquiring, analyzing, and managing data become key to data confidence and realizing business performance. FDA and industry could benefit from greater definition of adoption of standards around data management to clarify the core prerequisites for data hygiene, required to demonstrate data quality assurance. Educating reviewers and inspectors on the intricacies, pitfalls, and controls related to data management is essential to reduce the risk for both parties. The push to Artificial Intelligence (AI), Machine Learning (ML) and digital twins requires a clearly articulated framework that addresses development, validation, and quality elements from FDA, if FDA is to realize its objectives of promoting these new approaches for the benefit of all concerned.

## Leveraging Collaboration

The pandemic highlighted the extraordinary power of collaboration between health care providers, academia, industry, and regulators when addressing a common problem. Focused collaboration between industry, academia and FDA could clarify the routes to adoption for key concepts starting with surrogates to animal testing, AMT integration and shortening stability strategies.

## Conclusion

Innovation is an act of determination. To bring drug therapies to patients more quickly translates to an industry moving away from programs that will not be successful to allow them to focus on those that will. For the industry to become comfortable with adoption of AMTs and embrace complementary tools such as ASAP testing, digital twins, and other predictive models as a surrogate for traditional development activities means clarifying FDA’s expectations and concerns. FDA has already started the move toward recognizing the value of experience and expertise in evaluating the effectiveness of new drug therapies. Broadening these principles to leverage the power and effectiveness of new predictive approaches as a surrogate for real time data would be tremendous catalyst to improving the speed and effectiveness of the drug development lifecycle.
